# Salvianolic Acid Y: A New Protector of PC12 Cells against Hydrogen Peroxide-Induced Injury from *Salvia officinalis*

**DOI:** 10.3390/molecules20010683

**Published:** 2015-01-06

**Authors:** Jun Gong, Aichun Ju, Dazheng Zhou, Dekun Li, Wei Zhou, Wanli Geng, Bing Li, Li Li, Yanjie Liu, Ying He, Meizhen Song, Yunhua Wang, Zhengliang Ye, Ruichao Lin

**Affiliations:** 1 Department of Technology Development, TianJin Tasly Pride Pharmaceutical Co., Ltd., Tianjin 300410, China; E-Mails: gongjun_1986@126.com (J.G.); juach@tasly.com (A.J.); zhoudz@tasly.com (D.Z.); lidekun@tasly.com (D.L.); libing3@tasly.com (B.L.); lili4@tasly.com (L.L.); liuyanjie@tasly.com (Y.L.); heying@tasly.com (Y.H.); songmeizhen2013@tasly.com (M.S.); wangyunhua@tasly.com (Y.W.); 2 Tasly R&D Institute, Tasly Pharmaceutical Co., Ltd., Tianjin 300410, China; E-Mails: wzhou@tasly.com (W.Z.); 1122gengwanli@163.com (W.G.); 3 School of Chinese Materia Medica, Beijing University of Chinese Medicine, Beijing 100050, China

**Keywords:** PC12 cells, *Salvia officinalis*, circular dichroism (CD)

## Abstract

Salvianolic acid Y (TSL 1), a new phenolic acid with the same planar structure as salvianolic acid B, was isolated from *Salvia officinalis*. The structural elucidation and stereochemistry determination were achieved by spectroscopic and chemical methods, including 1D, 2D-NMR (^1^H-^1^H COSY, HMQC and HMBC) and circular dichroism (CD) experiments. The biosynthesis pathway of salvianolic acid B and salvianolic acid Y (TSL 1) was proposed based on structural analysis. The protection of PC12 cells from injury induced by H_2_O_2_ was assessed *in vitro* using a cell viability assay. Salvianolic acid Y (TSL 1) protected cells from injury by 54.2%, which was significantly higher than salvianolic acid B (35.2%).

## 1. Introduction

The dried root of *Salvia officinalis* (danshen) is a common traditional Chinese medicine widely used for the treatment of cardiovascular and cerebrovascular diseases, and its well-known antioxidant properties are attributed mainly to the presence of phenolic acids [1,2,3,4]. In the course of investigating water-soluble components of danshen, a new component was found, which is an epimeride of salvianolic acid B. Recent studies of the chemical constituents of danshen mainly focused on the hydrophilic compounds derived from caffeic acid, and more than twenty-five caffeic acid derivatives have been isolated and identified from the aqueous extracts of danshen, including salvianolic acid A–E, rosmarinic acid and lithospermic acid [5]. Many of these derivatives have significant pharmacological and biological activities [5].

Salvianolic acid B is the major component of danshen, and extensive pharmacological studies have been reported for this compound [6,7,8,9,10]. Primordially, salvianolic acid B was found in 1981 [11], and the configurational assignments were based on chemical degradation and circular dichroic correlation in 1988 [12]. The α,β-positions of the dihydrobenzofuran core were assigned the R/R-configuration. However, the absolute configurations previously assigned to the dihydrobenzofuran stereocenters of salvianolic acid B had been proven to be incorrect in 2006 [13] and were reassigned the S/S-configuration.

**Figure 1:**
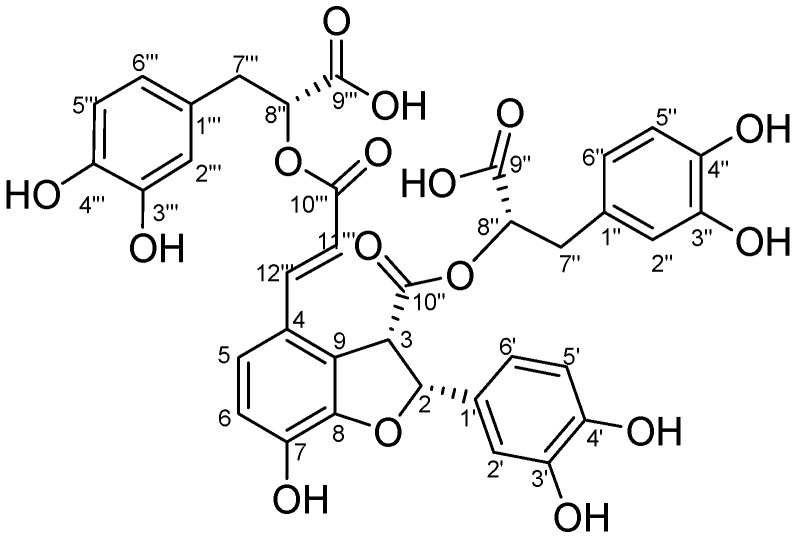
Structure of salvianolic acid Y (TSL 1).

Salvianolic acid Y (TSL 1) was isolated as a white powder from a water extract of danshen by repeated chromatography on a Sephadex LH-20 column, ODS column and preparative RP-HPLC. The compound was elucidated as 4-[(1*E*)-3-[(1*R*)-1-carboxy-2-(3,4-dihydroxphenyl)ethoxy]-3-oxo-1-propene-1-yl]-2-(3,4-dihydroxphenyl)-2*S*,3*S*-dihydro-7-hydroxy-, 3-[(1*R*)-1-carboxy-2-(3,4-dihydroxphenyl)ethyl] ester by NMR and CD (Figure 1). Its ^1^H-NMR and ^13^C-NMR chemical shifts were similar to those of salvianolic acid B (Table 1).

**Table 1:** NMR data of salvianolic acid Y (TSL 1) and salvianolic acid B (methanol-*d*_4_, 400 MHz/100 MHz).

Position	TSL 1		Salvianolic Acid B	
	δ_H_ (ppm)	δc (ppm)	δ_H_ (ppm)	δc (ppm)
2	5.90 (d,9.2)	87.0	5.81 (d,4.8)	86.7
3	4.77 (d,9.2)	53.2	4.30 (d,4.8)	56.4
4	-	123.2	-	123.1
5	7.11 (d, 8.5)	121.3	7.11 (d, 8.5)	120.6
6	6.77 (d, 8.4)	116.9	6.78 (d, 8.4)	116.7
7	-	145.4	-	145.1
8	-	148.2	-	147.5
9	-	126.8	-	123.1
1'	-	127.8	-	127.7
2'	6.90 (s)	113.6	6.69	111.8
3'	-	144.4	-	144.2
4'	-	143.9	-	143.6
5'	6.68 (ov)	114.7	6.70 (ov)	114.8
6'	6.68 (ov)	118.4	6.70 (ov	116.8
1''	-	126.9	-	127.4
2''	6.48 (d,1.9)	116.4	6.47 (d,1.9)	115.7
3''	-	143.8	-	143.4
4''	-	143.7	-	143.4
5''	6.56 (d,8.0)	114.9	6.49 (d,8.0)	114.9
6''	6.33 (dd,8.1,1.9)	120.7	6.26 (dd,8.0,1.9)	120.1
7''α	2.54 (dd,14.0,6.1)	36.1	2.94 (ov)	35.9
7''β	2.45 (dd,14.0,6.6)	-	2.78 (dd,16.0,8.0)	-
8''	4.35 (t, 6.3)	74.4	5.11 (t, 4.0)	74.1
9''	-	171.0	-	171.1
10''	-	170.0	-	170.7
1'''	-	127.7	-	127.7
2'''	6.71 (d,1.9)	116.4	6.70 (d,1.9)	115.7
3'''	-	144.8	-	145.0
4'''	-	144.6	-	144.3
5'''	6.64 (d, 8.0)	114.9	6.65 (d, 8.0)	114.8
6'''	6.57 (dd, 8.0, 1.9)	120.8	6.57 (dd, 8.0, 1.9)	120.1
7'''α	3.02 (dd,14.3,4.8)	36.4	3.03 (dd,16.0,4.0)	36.3
7'''β	2.98 (dd,14.2,6.9)	-	2.95 (ov)	-
8'''	5.11 (t, 6.7)	73.3	5.11 (t, 4.0)	73.2
9'''	-	171.2	-	172.2
10'''	-	166.7	-	166.4
11'''	6. 25 (d,16.0)	115.5	6. 15 (d,16.0)	115.0
12'''	7.53 (d, 16.0)	142.4	7.46 (d, 16.0)	141.9

δ in ppm; ov = overlapped.

## 2. Results and Discussion

### 2.1. Structural Elucidation of Salvianolic Acid Y

Salvianolic acid Y (TSL 1) was obtained as an optically-active, white, amorphous powder, ${\left[\alpha \right]}_{D}^{10}$–42° (*c* 0.87, CH_3_OH). The molecular formula was determined as C_36_H_30_O_16_ by the HRESI-MS negative ion peak at *m/z* 717.1338 [M−H]^−^, with 22 degrees of unsaturation. It showed a positive reaction with 1% FeCl_3_ test solution. The UV spectrum displayed absorptions at 207, 255, 289 and 307 nm, almost in accordance with the reported UV absorptions of salvianolic acids B [12]. The IR spectrum indicated the presence of hydroxyl (3357 cm^−1^), carbonyl (1721 cm^−1^) and aromatic ring (1611, 1527, 1448 cm^−1^) functionalities in the structure of salvianolic acid Y (TSL 1). The ^1^H-NMR spectrum of salvianolic acid Y presented fifteen downfield proton signals, including three sets of ABX coupling system protons (I: δ 6.71 (1H, d, 1.9, H-2'''), δ 6.64 (1H, d, 8.0, H-5''') and δ 6.57 (1H, dd, 8.0, 1.9, H-6'''); II: δ 6.48 (1H, d, 1.9, H-2''), δ 6.56 (1H, d, 8.0, H-5'') and δ 6.33 (1H, dd, 8.1, 1.9, H-6''); III: δ 6.90 (1H, s, H-2') and δ 6.68 (2H, ov, H-5'/H-6')), two sets of AB coupling system proton signals (δ 7.11 (1H, d, 8.5, H-5) and δ 6.77 (1H, d, 8.4, H-6); δ 5.90 (1H, d, 9.2, H-2) and δ 4.77 (1H, d, 8.0, H-3)), together with two singlet aromatic proton signals at δ 6.25 (1H, d, 16.0, H-11'''), δ 7.53 (1H, d, 16.0, H-12'''). There still existed two sets of AX_2_ coupling protons (I: δ 4.35 (1H, t, 6.3, H-8''), 2.54 (1H, dd, 14.0, 6.1, H-7''α), 2.45 (1H, dd, 14.0, 6.6, H-7''β); II: δ 5.11 (1H, t, 6.7, H-8'''), 3.02 (1H, dd, 14.3, 4.8, H-7'''α), 2.98 (1H, dd, 14.2, 6.9, H-7'''β). The ^13^C-NMR spectrum exhibited 36 signals, of which two carbons were methylenes, 17 were methines and quaternary carbons, as shown in the DEPT spectrum. Further analysis demonstrated four carbonyl signals at δ 171.0 (COOH-9'' ), δ 170.0 (COO-10''), δ 171.2 (COOH-9''') and δ 166.7 (COO-10'''); 13 quaternary aromatic carbons at δ 123.2 (C-4), 145.4 (C-7), 148.2 (C-8), 126.8 (C-9), 127.8 (C-1'), 144.4 (C-3'), 143.9 (C-4'), 126.9 (C-1''), 143.8 (C-3''), 143.7 (C-4''), 127.7 (C-1'''), 144.8 (C-3''') and 144.6 (C-4'''); 17 methine carbons at δ 87.0 (C-2), 53.2 (C-3), 121.3 (C-5), 116.9 (C-6), 113.4 (C-2'), 114.7(C-5'), 118.4 (C-6'), 116.4 (C-2'') , 114.9 (C-5''), 120.7 (C-6''), 74.4 (C-8''), 116.4 (C-2'''), 114.9 (C-5'''), 120.8 (C-6'''), 73.3 (C-8'''), 115.5 (C-11''') and 142.4 (C-12'''); as well as two methylene carbons at δ 36.1 (C-7'') and 36.4 (C-7'''); as shown in Table 1.

**Figure 2:**
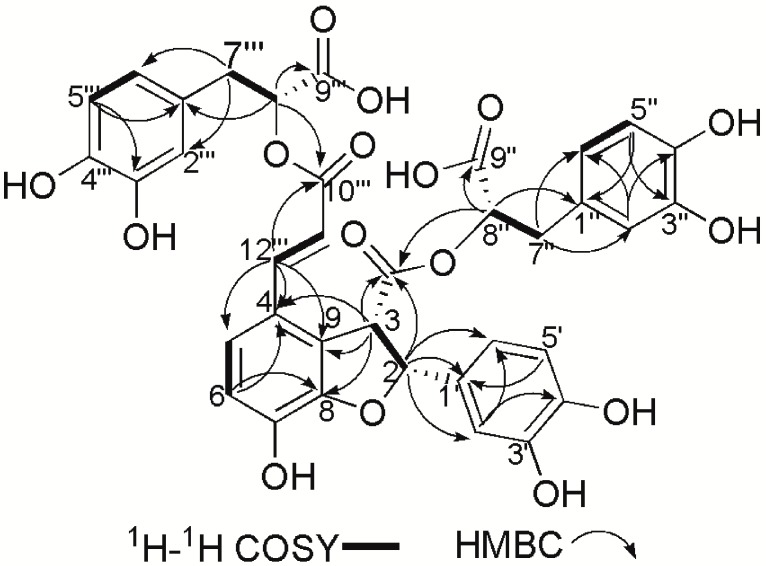
Key ^1^H-^1^H COSY and HMBC correlations (H→C) in TSL 1.

The HMBC spectrum presented correlation signals from δ 5.90 (H-2) to 127.8 (C-1')/113.4 (C-2')/118.4 (C-6')/53.2 (C-3)/170.0 (C-10''), from δ 4.77 (H-3) to 87.0 (C-2)/170.0 (C-10'')/148.2 (C-8)/126.8 (C-9)/123.2 (C-4), from δ 4.35 (H-8'') to 171.0 (C-9'')/170.0 (C-10'')/36.1 (C-7'')/126.9 (C-1''), from δ 2.54 (H-7''α), 2.45 (H-7''β) to 74.4 (C-8'')/171.0 (C-9'')/120.7 (C-6'')/116.4 (C-2'')/126.9 (C-1''), from δ 6.48 (H-2'') to 36.1 (C-7'')/ 120.7 (C-6'')/143.8 (C-3'')/ 143.7 (C-4''), from δ 7.53 (H-12''') to 123.2 (C-4)/121.3 (C-5)/126.8 (C-9)/115.5 (C-11''')/166.7 (C-10'''), from δ 6.25 (H-11''') to 123.2 (C-4)/166.7 (C-10'''), from δ 5.11 (H-8''') to 166.7 (C-10''')/171.2 (C-9''')/36.4 (C-7''')/127.7 (C-1'''), from δ 3.02 (H-7'''α), 2.98 (H-7'''β) to 73.3 (C-8''')/171.0 (C-9''')/127.7 (C-1''')/116.4 (C-2''')/120.8 (C-6''') and from δ 6.77 (H-6) to 123.2 (C-4)/148.2 (C-8) (Figure 2). Moreover, the ^1^H-^1^H COSY and HMQC spectra indicated the presence of such structural units as CH (H-2)-CH (H-3), CH (H-5)=CH (H-6), CH (H-11''')=CH (H-12'''), CH (H-5'')=CH (H-6''), CH (H-8'')-CH_2_(H-7''), CH (H-8''')-CH_2_(H-7''') and CH (H-5''')=CH (H-6'''). Based on the combined 1D-, 2D-NMR spectral data discussed above, the planar structure of the compound was identified.

The coupling constants between H-2 and H-3(*J* = 9.2 Hz) suggested that they were *cis*-related (2*S*,3*R* or 2*R*,3*S*) [14,15], which was different from the *trans*-related (2*S*,3*S*)-configuration of salvianolic acid B [13].

**Figure 3:**
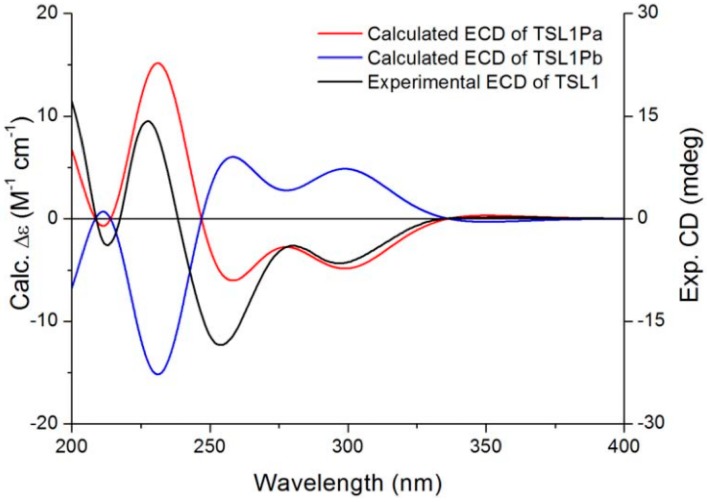
Experimental solution spectrum (black) of salvianolic acid Y (TSL 1) compared with the calculated ECD spectra of (2*R*,3*S*)-TSL 1Pa (red) and (2*S*,3*R*)-TSL 1Pb (blue).

The absolute configuration of salvianolic acid Y (TSL 1) was assigned by the time-dependent density functional theory (TDDFT) ECD calculation protocol [16,17,18], a powerful method for the configurational assignment of natural products. The optimized conformations were used for the ECD calculations, which were performed with Gaussian 09 (B3LYP/6-31G(d)). The calculated ECD spectra of salvianolic acid Y were compared with the experimental ECD spectrum to determine the most probable configuration. Although, as shown in Figure 3, the calculated absorption wavelengths did not fit exactly with the experimental peak positions, the differences between the calculated and experimental spectra presumably resulted from an overestimation of the UV absorbance in the calculations or may be due to minor differences between calculated and solution conformers [18,19,20]. Finally, the Boltzmann-averaged ECD spectra of (2*R*,3*S*)-salvianolic acid Y (TSL 1Pa) was in agreement with the experimental one, allowing the determination of the absolute configuration of salvianolic acid Y as (2*R*,3*S*) (Figure 3).

### 2.2. In Vitro Biological Activity

The protection activity of salvianolic acid B and salvianolic acid Y on hydrogen peroxide (H_2_O_2_)-induced cell lesion was investigated in rat pheochromocytoma line PC12 [21,22,23]. According to the respective calculations, it was shown that the protection rates of salvianolic acid B and salvianolic acid Y were 35.2% and 54.2%, respectively (Table 2). Apparently, salvianolic acid Y protected cell injury by 54.2%, which was significantly higher than salvianolic acid B (35.2%).

**Table 2:** Salvianolic acid B and salvianolic acid Y protect PC12 cells against H_2_O_2_-induced injury.

Group	Dose (μM)	Cell Viability (%)	Protection Rate (%)
control group	-	100 ± 6.5	-
model group	-	49.3 ± 6.0 ^##^	-
salvianolic acid B	10	68.7 ± 3.0 **	35.2 ± 6.2
salvianolic acid Y	10	79.2 ± 2.6 **	54.2 ± 5.4 ^@@^

*x* ± *s*, *n* = 10; ^##^ *p* < 0.01 compared with the control group; ** *p* < 0.01, compared with the model group; ^@@^ *p* < 0.01, compared with the salvianolic acid B group.

## 3. Experimental Section

### 3.1. General

Optical rotations were measured with a Perkin-Elmer 241 Polarimeter. UV absorption spectra were recorded on a Varian Cary 100 UV-vis spectrophotometer (Varian, Santa Clara, CA, USA). IR spectra were recorded on a Nicolet 5700 FTIR spectrometer (Thermo Scientific, Waltham, MA, USA); peaks are reported in cm^−1^. ECD spectra were measured on a Jasco J-715 spectropolarimeter (JASCO, Easton, MD, USA). The NMR spectra were recorded at 300 K on a Bruker Avance 400 spectrometer (Bruker, Billeria, MA, USA). Chemical shifts are given in parts per million (δ) with solvent (methanol-*d*_4_) and with TMS as the internal standard, assignments were supported by COSY, HMQC, HMBC and NOESY experiments. ESIMS were measured on an Agilent 6320 Ion Trap mass spectrometer (Agilent, Santa Clara, CA, USA). HRESIMS were measured on an Agilent 6210 TOF mass spectrometer (Agilent, Santa Clara, CA, USA). Sephadex LH-20 (Amersham Pharmacia Biotech AB, Uppsala, Sweden) and ODS (45–70 µm, Fuji Silysia, Kasugai, Japan) were used for column chromatography. Semipreparative HPLC was carried out on a BUCHI instrument equipped with a UV photometer (BUCHI, Flawil, Switzerland), employing a Venusil-XBP-C_18_ column (50 × 250 mm, 10 μm).

### 3.2. Compound Isolation

The danshen extract (lyophilized powder) (2.2 g) was firstly dissolved in water and then subjected to Sephadex LH-20 column chromatography eluted by MeOH–H_2_O (2:1) to afford five fractions (Fr. 1–5). Fr. 4 (271.8 mg) was subsequently chromatographed over the ODS column using a gradient of EtOH–H_2_O (10:90, 30:70, 60:40 and 95:5) as the eluent to give four fractions (Fr. 4A–4D). Finally, salvianolic acid Y (31.8 mg) was obtained by preparative RP-HPLC with a gradient of CH_3_CN–0.05%CF_3_COOH (10:90, 22:78, 26:74, 39:61, v/v) as the eluting mobile phase.

### 3.3. Activity in Vitro

Rat pheochromocytoma PC12 cells, purchased from Shanghai Institute of Cell Biology, Chinese Academy of Sciences, were grown in tissue culture flasks in DMEM supplemented with 10% fetal bovine serum, and 100 U/mL penicillin and 100 U/mL streptomycin. The cultures were maintained at 37 °C in a humidified atmosphere containing 5% CO_2_. The culture media were changed every 2 days. The cells were inoculated to 96-well plates when grown to anastomose and treated with 10 µM salvianolic acid B and 10 µM salvianolic acid Y for 6 h, respectively, followed by co-culture with 400 μM H_2_O_2_ for another 1 h.

Cell survival was evaluated by the method of MTT (3-(4,5-dimethylthiazol-2-yl)-2,5-diphenyltetra-zolium bromide). The OD value was measured at λ = 570 nm by a spectrophotometer.

The protection rate of tested compounds was calculated using the following equation:

protection rate (%) = (cell viability of drug group − cell viability of model group)/(cell viability of control group − cell viability of model group) × 100%

### 3.4. Statistical Analysis

All *in vitro* biological activity tests were repeated at least twice independently, and the measurements in each experiment were run in triplicate. Results are presented as the means ± SD. Statistical significance was determined using one-way ANOVA, followed by the Student’s two-tailed *t*-test for comparison between two groups or Dunnett’s test when the data involved three or more groups. *p* < 0.05 was considered to be significant.

### 3.5. Biosynthetic Pathway 

**Scheme 1:**
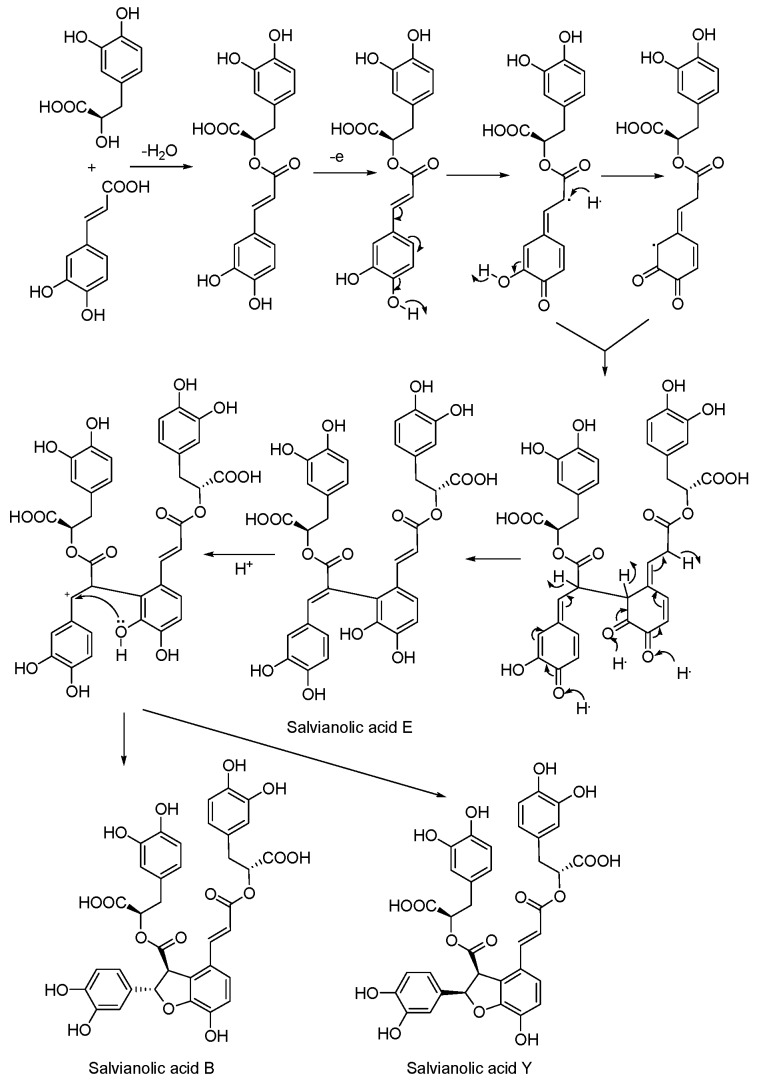
Plausible biosynthetic pathway of salvianolic acid E, B and Y.

We proposed a plausible biogenetic pathway for salvianolic acid Y at the theory level, as shown in Scheme 1.

## 4. Conclusions

A new phenolic acid with the same planar structure as salvianolic acid B, 4-[(1*E*)-3-[(1*R*)-1-carboxy-2-(3,4-dihydroxphenyl)ethoxy]-3-oxo-1-propene-1-yl]-2-(3,4-dihydroxphenyl)-2*S*,3*S*-dihydro-7-hydroxy-, 3-[(1*R*)-1-carboxy-2-(3,4-dihydroxphenyl)ethyl] ester, was isolated from the extract of *Salvia officinalis*. The structural elucidation and stereochemistry determination were achieved by spectroscopic and chemical methods. The new compound was named salvianolic acid Y. For the *In vitro* biological activity test, our results suggest showed that salvianolic acid Y could rescue cell injury by H_2_O_2_, which was significantly better than salvianolic acid B. A plausible biogenetic pathway for salvianolic acid Y was proposed by our study.

## Supplementary Materials

Supplementary materials can be accessed at: http://www.mdpi.com/1420-3049/20/01/0683/s1.

## References

[1] Lu Y.R., Foo L.Y. (2002). Polyphenolics of Salvia—A review. Phytochemistry.

[2] Liu J., Dai Z., Wang G.L., Lin R.C. (2012). Progress in bioactive constituents and isolation and analysis method of *Salvia miltiorrhizae radix* et ehizoma. Chin. J. Exp. Trad. Med. Form..

[3] Jiang R.W., Lau K., Hon P.M., Mark T.C.W., Woo K.S., Fung K.P. (2005). Chemistry and biological activities of caffeic acid derivatives from *Salvia miltiorrhiza*. Curr. Med. Chem..

[4] Chang H.M., Cheng K.P., Choang T.F., Chow H.F., Chui K.Y., Hon P.M., Tan F.W.L., Zhong Z.P., Lee C.M., Sham H.L. (1990). Structure elucidation and total synthesis of new tanshinones isolated from *Salvia miltiorrhizae Bunge* (Danshen). J. Org. Chem..

[5] Li L.N. (1997). Water Soluble Active Components of *Salvia miltiorrhiza* and Related Plants. J. Chin. Pharm. Sci..

[6] Zan Y.E., Xu J.P. (2007). Advances in pharmacological effects of salvianolic acid B. Mil. Med. J. S. Chin..

[7] Zhao G.R., Zhang H.M., Ye T.X., Xiang Z.J., Yuan Y.J., Guo Z.X., Zhao L.B. (2008). Characterization of the radical scavenging and antioxidant activities of danshensu and salvianolic acid B. Food Chem. Toxicol..

[8] Cheng B., Gong H., Li X.C., Sun Y., Chen H., Zhang X., Wu Q., Zheng L., Huang K. (2013). Salvianolic acid B inhibits the amyloid formation of human islet amyloid polypeptideand protects pancreatic β-cells against cytotoxicity. Proteins Struct. Funct. Bioinform..

[9] Liu M., Ye J.T., Gao S., Fang W., Li H., Geng B., Zou J., Chen X., Chen S.R., Zhang L.K. (2014). Salvianolic acid B protects cardiomyocytes from angiotensin II-induced hypertrophy via inhibition of PARP-1. Biochem. Biophys. Res. Commun..

[10] Xu D.H., Xu L.L, Zhou C.H., Wayne Y.W. Lee, Wu T., Cui L., Li G. (2014). Salvianolic acid B promotes osteogenesis of human mesenchymal stem cells through activating ERK signaling pathway. Int. J. Biochem. Cell Biol..

[11] Chen Z.X., Gu W.H., Huang H.Z., Yang X.M., Sun C.J., Chen W.Z., Dong Y.L., Ma H.L. (1981). Studies of water-soluble phenolic acid components of Salvia. Chin. Pharm. Bull..

[12] Ai C.B, Li L.N. (1988). Stereostructure of salvianolic acid B and isolation of salvianolic acid C from *Salvia miltiorrhiza*. J. Nat. Prod..

[13] Anja W., Steven J.O., Robert G.B., Jonathan A.E. (2006). Reassignment of the configuration of salvianolic acid B and establishment of its identity with lithospermic acid B. J. Nat. Prod..

[14] Wada H., Kido T., Tanaka N., Murakami T., Saiki Y., Chen C.M. (1992). Chemical and chemotaxonomical studies of ferns. LXXXI. Characteristic lignans of Blechnaceous ferns. Chem. Pharm. Bull..

[15] Zhang Z.F., Peng Z.G., Gao L., Dong B., Li J.R., Li Z.Y., Chen H.S. (2008). Three new derivatives of anti-HIV-1 polyphenols isolated from *Salvia yunnanensis*. J. Asian Nat. Prod. Res..

[16] Nina B., Lorenzo D.B., Gennaro P. (2007). Application of electronic circular dichroism in configurational and conformational analysis of organic compounds. Chem. Soc. Rev..

[17] Zou Y., Mark T.H. (2013). Atkamine: A New Pyrroloiminoquinone Scaffold from the Cold Water Aleutian Islands *Latrunculia* Sponge. Org. Lett..

[18] Marialuisa M., Anna A., Filomena D., Concetta I., Paolo L., Rocco V., Carlo I., Rita S. (2013). Conithiaquinones A and B, tetracyclic cytotoxic meroterpenes from the mediterranean Ascidian *Aplidium conicum*. Eur. J. Org. Chem..

[19] Haidy N.K., Ding Y.Q., Li X.C., Daneel F., Frank R.F., Marc S. (2009). Beyond Polymaxenolide: Cembrane-Africanane terpenoids from the Hybrid Soft Coral *Sinularia maxima* × *S. polydactyla*. J. Nat. Prod..

[20] Wilanfranco C.T., Miho H., Saki K., Shogo N., Kazuaki T., Tatsuo N., Masaru H. (2011). Stereochemical investigations of isochromenones and isobenzofuranones isolated from *Leptosphaeria* sp. KTC 727. J. Nat. Prod..

[21] Xiao X.Q., Yang J.W., Tang X.C. (1999). Huperzine A protects rat pheochromocytoma cells against hydrogen peroxide-induced injury. Neurosci. Lett..

[22] Zhang H.Y., Tang X.C. (2000). Huperzine B, a novel acetylcholinesterase inhibitor, attenuates hydrogen peroxide induced injury in PC12 cells. Neurosci. Lett..

[23] Li S.P., Zhao K.J., Ji Z.N., Song Z.H., Dong T.T.X., Lo C.K., Cheung J.K.H., Zhu S.Q., Tsim K.W.K. (2003). A polysaccharide isolated from *Cordyceps sinensis*, a traditional Chinese medicine, protects PC12 cells against hydrogen peroxide-induced injury. Life Sci..

