# Supplementary material for: Salvianolic Acid Y: A New Protector of PC12 Cells against Hydrogen Peroxide-Induced Injury from Salvia officinalis
Source: Molecules. 2015 Jan 6;20(1):683–92. doi: 10.3390/molecules20010683 (PMC6272257; doi:10.3390/molecules20010683)
Supplement: Supplementary file 1 [file molecules-20-00683-s001.pdf]

Supplementary Materials

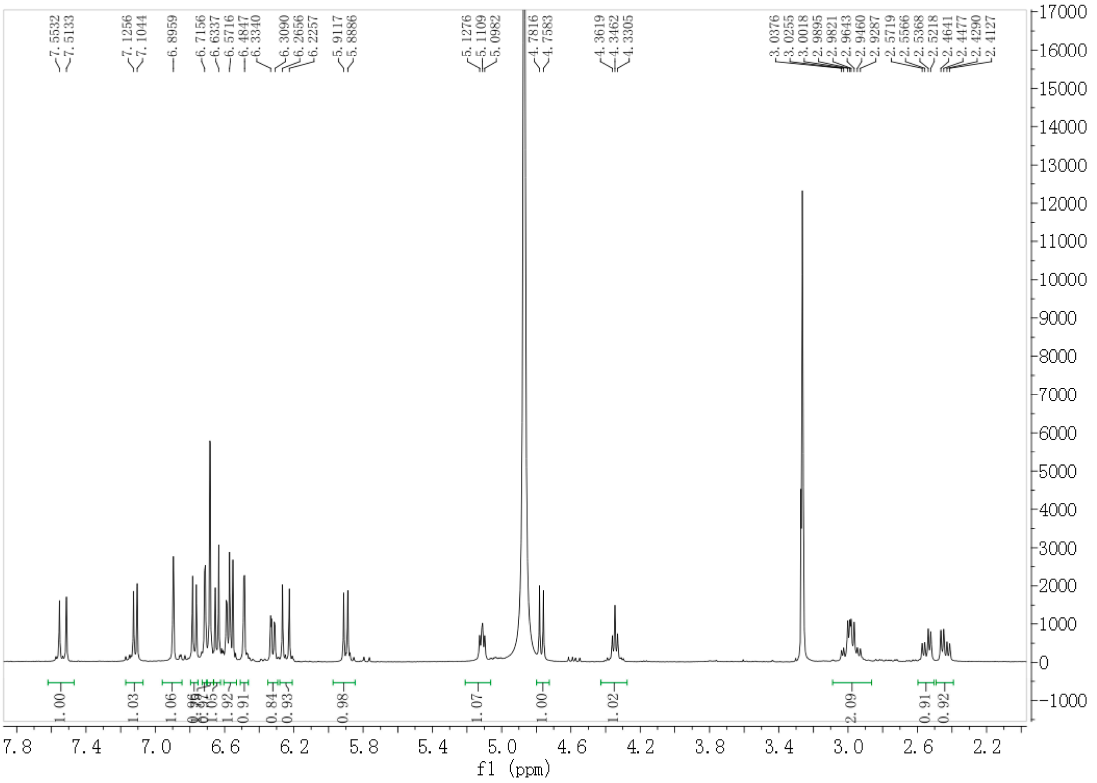

Figure S1. ^{1}H-NMR spectrum of salvianolic acid Y (TSL 1).

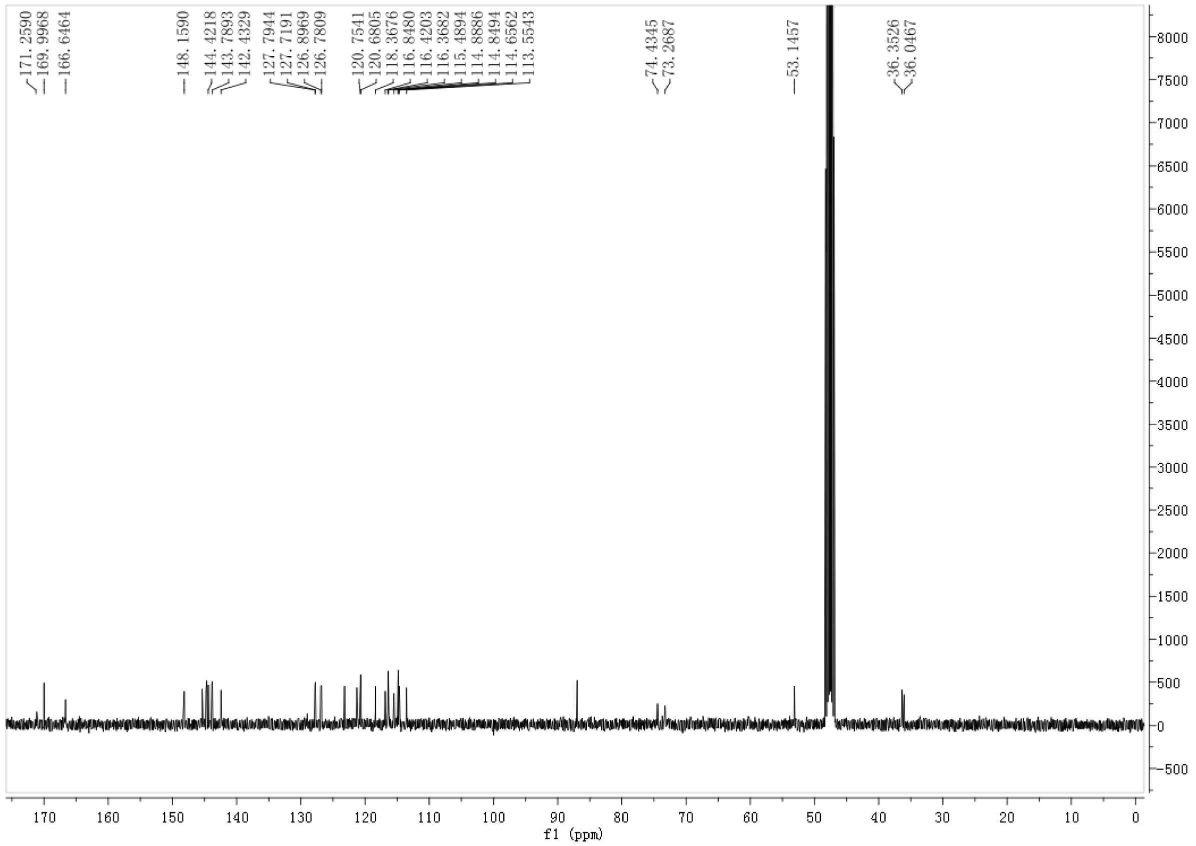

Figure S2. ^{13}C-NMR spectrum of salvianolic acid Y (TSL 1).

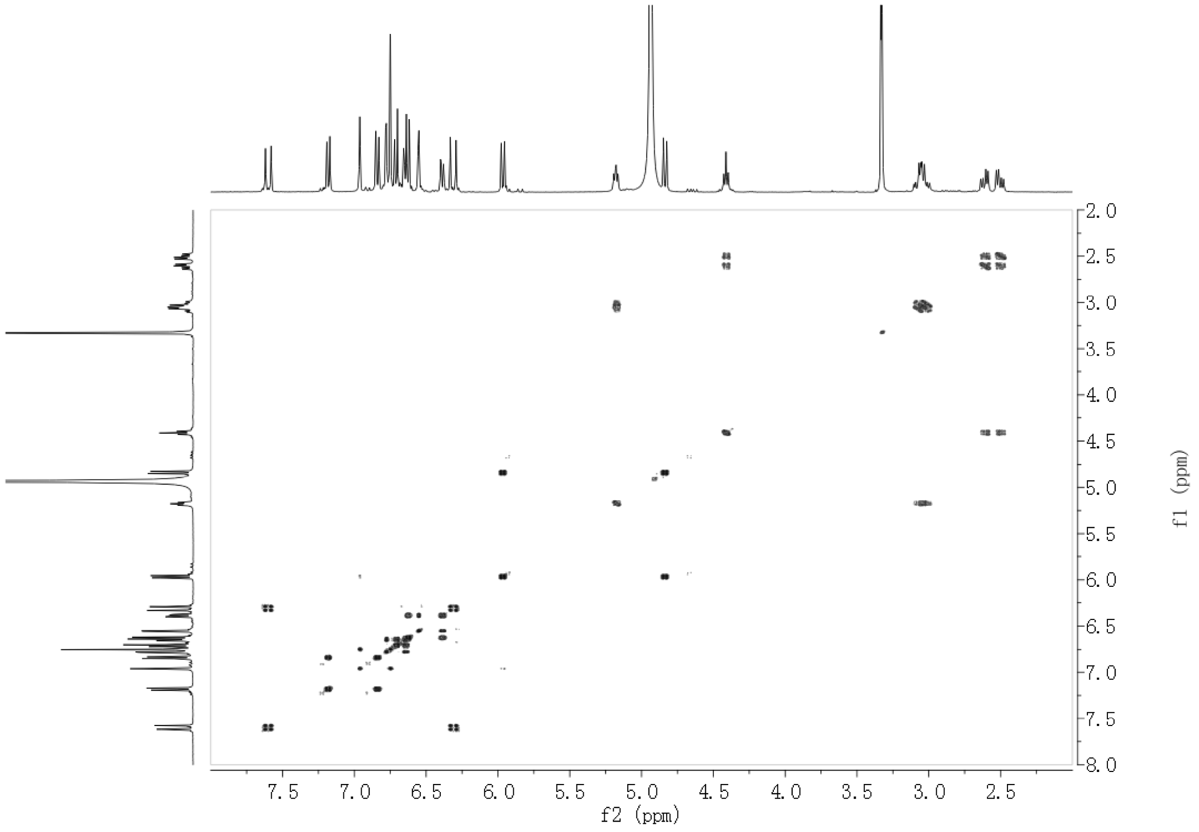

Figure S3. ^{1}H-^{1}H COSY spectrum of salvianolic acid Y (TSL 1).

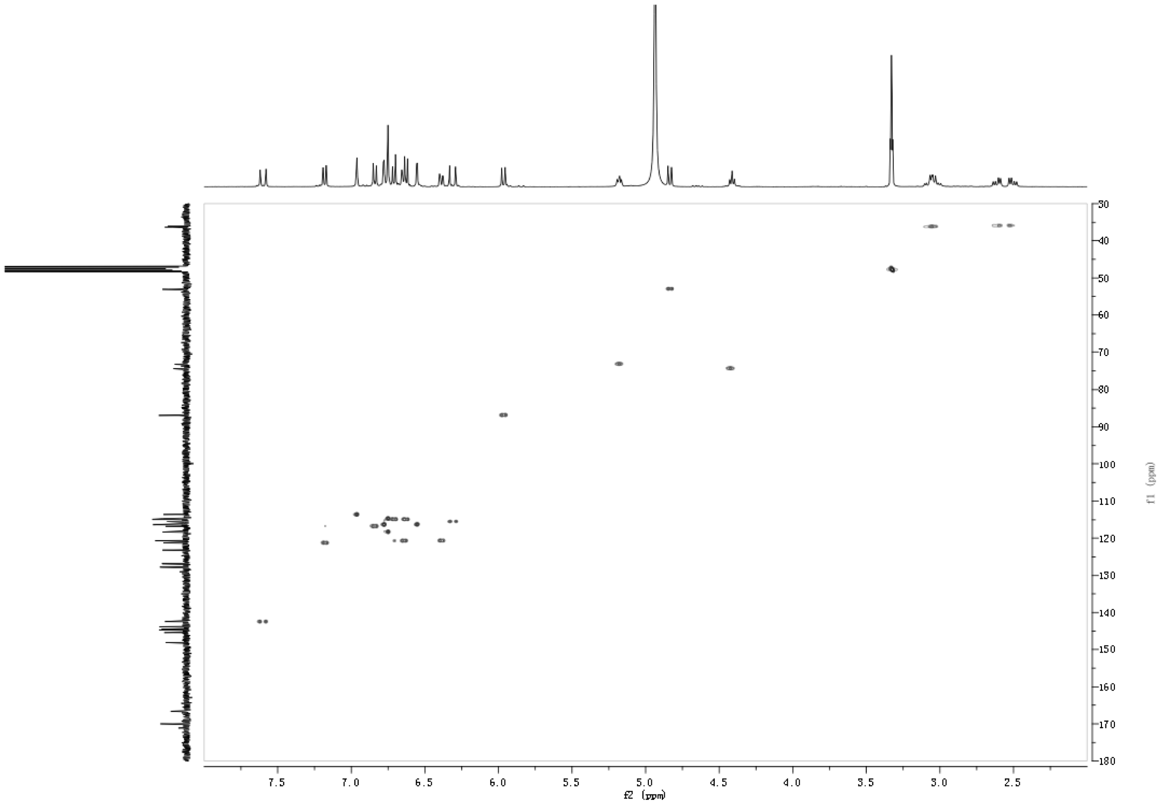

Figure S4. HMQC spectrum of salvianolic acid Y (TSL 1).

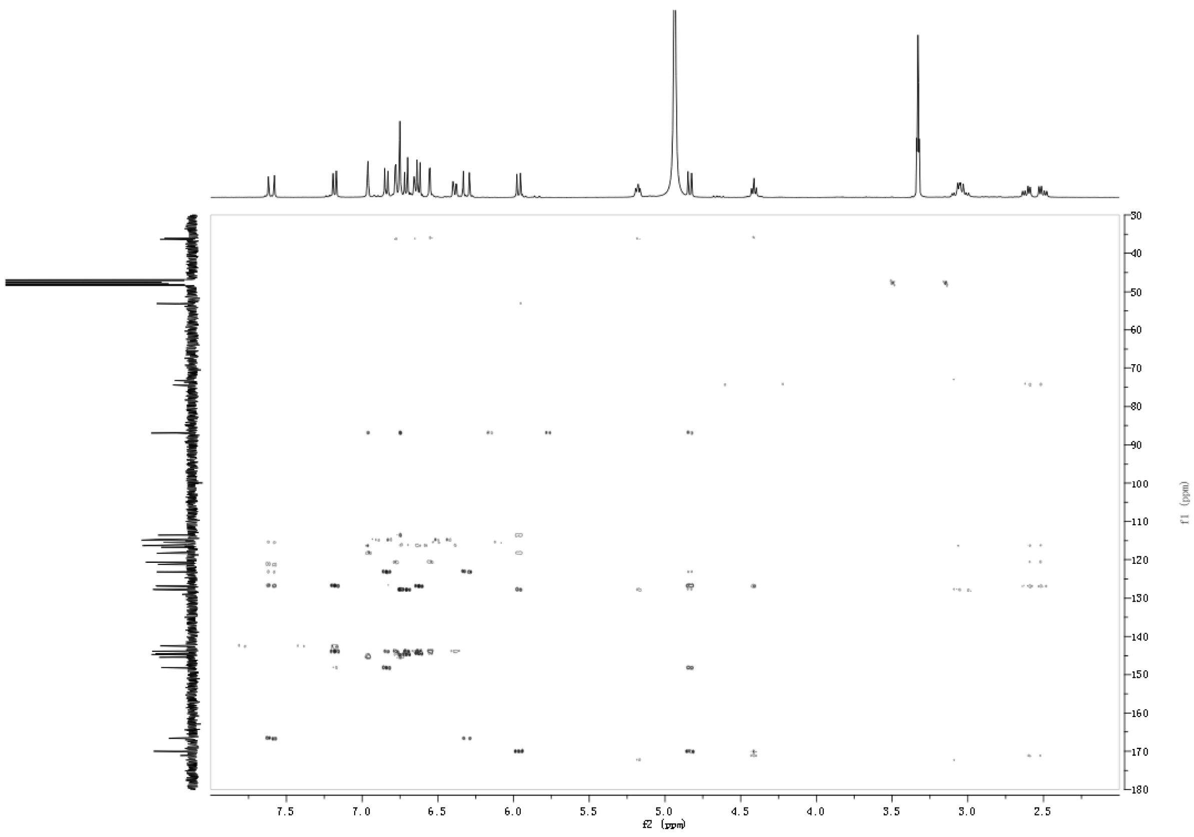

Figure S5. HMBC spectrum of salvianolic acid Y (TSL 1).

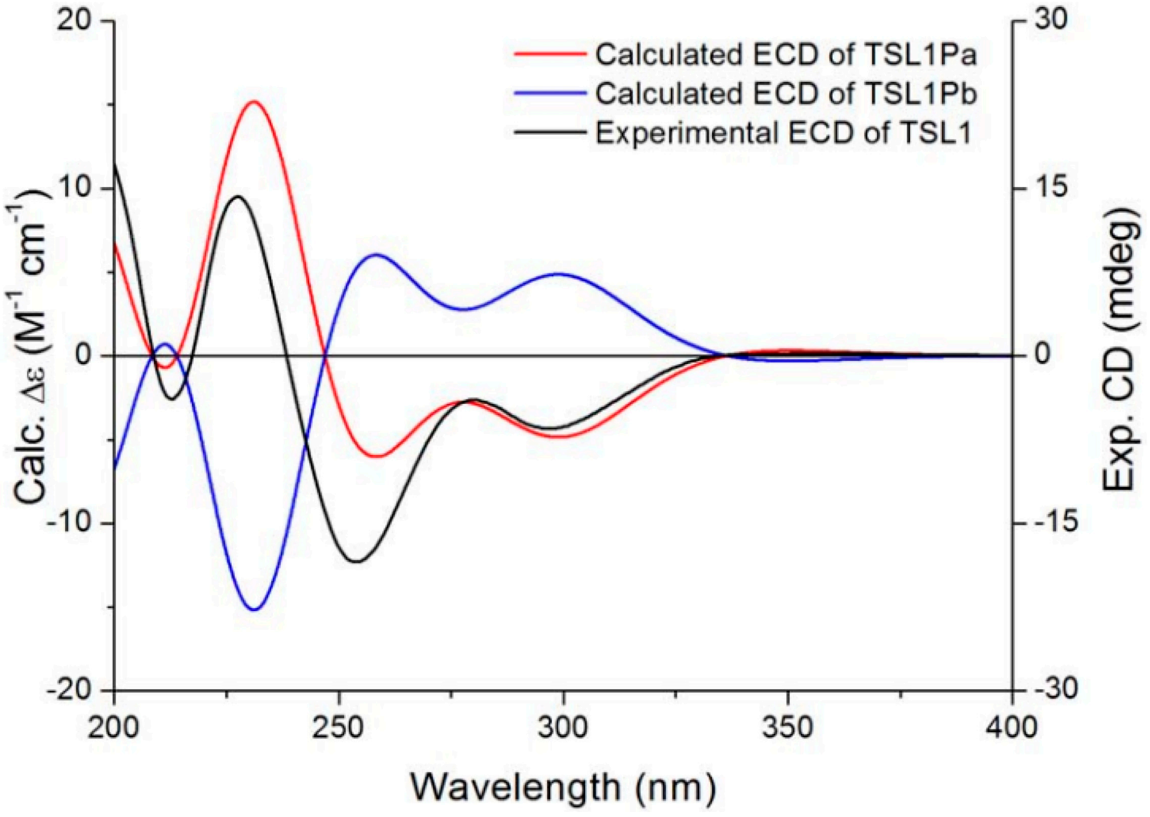

Figure S6. Experimental solution spectrum **(**black) of salvianolic acid Y (TSL 1) compared with the calculated ECD spectra of (2*R*,3*S*)-TSL 1Pa (red) and (2*S*,3*R*)-TSL 1Pb (blue).

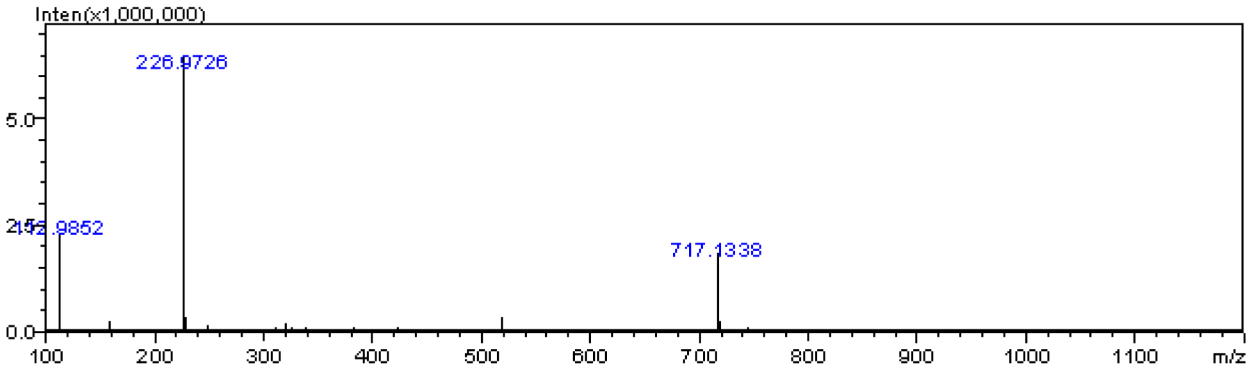

Figure S7. HR-ESI-MS spectrum of salvianolic acid Y (TSL 1).
